# Editorial: Autoimmunity: novel insights and future perspectives

**DOI:** 10.3389/fimmu.2025.1728233

**Published:** 2025-10-31

**Authors:** Monica Neagu, Mihaela Adriana Ilie, Ancuta Mihai, Constantin Caruntu

**Affiliations:** ^1^ Immunology Department, “Victor Babes” National Institute, Bucharest, Romania; ^2^ Pathology Department, Colentina Clinical Hospital, Bucharest, Romania; ^3^ Faculty of Biology, University of Bucharest, Bucharest, Romania; ^4^ Dermatology Department, Kalmar County Hospital, Kalmar, Sweden; ^5^ Internal Medicine - Rheumatology Department, Carol Davila Central Military Emergency University Hospital, Bucharest, Romania; ^6^ Faculty of Medicine, Titu Maiorescu University, Bucharest, Romania; ^7^ Réseau de l’arc, Hôpital de Moutier, Berne, Switzerland; ^8^ Department of Physiology, The “Carol Davila” University of Medicine and Pharmacy, Bucharest, Romania; ^9^ Department of Dermatology, “Prof. N.C. Paulescu, National Institute of Diabetes, Nutrition and Metabolic Diseases, Bucharest, Romania

**Keywords:** autoimmune diseases, autoantibodies, immunoregulatory proteins, biomarkers, diagnosis, immunomodulatory therapies

This Research Topic on autoimmunity was a success, attracting the attention of many research teams worldwide and resulting in no fewer than 40 articles, of which 60% were original studies. The work presented focuses on rheumatoid arthritis, psoriatic arthritis, psoriasis, lupus, and several other autoimmune diseases and autoimmune-related conditions. As recent large cohort studies have shown that one in 10 individuals is diagnosed with an autoimmune disease, and their incidence is rising, especially in Western countries (1, 2), this Research Topic gathers the most recent original studies and review articles on this important human condition.

Autoimmune diseases represent a complex and heterogeneous group of disorders characterized by immune dysregulation, chronic inflammation, and multiorgan involvement (2–8). Over the past decades, advances in immunology, genetics, and systems biology have deepened our understanding of the mechanisms underlying these diseases (4–17), while simultaneously uncovering novel biomarkers and therapeutic targets (4, 18–28).

This Research Topic brings together cutting-edge studies that collectively provide new insights into the pathogenesis, systemic consequences, and emerging treatment strategies for autoimmune diseases, with a focus on rheumatoid arthritis (RA), psoriatic arthritis (PsA), ankylosing spondylitis (AS), psoriasis (PsO), and antiphospholipid syndrome (APS). By integrating insights from immunology, microbiology, proteomics, genetics, and innovative modeling platforms, these articles offer multidimensional perspectives on autoimmunity.

The majority of the contributions focus on rheumatoid arthritis and psoriatic arthritis. A central theme in this Research Topic is immune dysregulation as a driver of disease (9, 29–31). Yan et al. explored the immunological landscape of difficult-to-treat RA (D2T RA), revealing a marked reduction in regulatory T cells (Tregs) accompanied by an increased Th17/Treg ratio, reflecting a disrupted immune balance that correlates with heightened disease activity. Importantly, low-dose interleukin-2 therapy successfully restored Treg populations without significant adverse effects, highlighting the potential of targeted immunomodulation to re-establish immune homeostasis in refractory RA. This study emphasizes the pivotal role of Tregs as a hallmark of severe disease and underscores the therapeutic promise of restoring immune equilibrium rather than broadly suppressing inflammation.

Complement activation is another dimension of immune dysregulation that is particularly relevant in systemic autoimmune pathologies (32–35). Erkan et al. investigated complement activation in patients positive for antiphospholipid antibodies (aPLs) who did not have other systemic autoimmune rheumatic diseases. They demonstrated that cell-bound complement activation products (CB-CAPs) on B lymphocytes, erythrocytes, and platelets are more sensitive indicators of complement activity than traditional C3/C4 measurements. Elevated CB-CAPs were most pronounced in patients with microvascular APS, thrombocytopenia, or hemolytic anemia, and they remained stable over 6 to 12 months. These findings suggest that CB-CAPs could serve as reliable biomarkers for monitoring disease activity and thrombosis risk, providing a tool for more precise clinical management of APS, where conventional complement measures may underestimate activation. Feng et al. highlighted the crucial role of endothelial cells (ECs) in the pathogenesis of APS, an autoimmune disease associated with recurrent thromboses and recurrent pregnancy losses (36–42). Antiphospholipid antibodies (aPLs) were found to act as both biomarkers and pathogenic factors, directly interacting with EC receptors and activating intracellular pathways involved in various pathophysiological mechanisms (37, 43–47). *In vitro* and *in vivo* studies have described multiple molecular mechanisms through which ECs mediate the effects of aPLs on vascular function (48–50). These findings provide an integrated understanding of the role of ECs in APS and identify new potential targets for diagnosis and the development of personalized therapies.

Wang et al. explored the role of the ubiquitin-proteasome system (UPS) in the pathogenesis of APS, an autoimmune disease characterized by thromboses and pregnancy complications associated with persistent elevation of aPLs (51). UPS imbalance promotes the activation of proinflammatory and prothrombotic pathways, contributing to disease progression (52, 53). The summarized *in vivo* studies presented in their work suggest that low-dose proteasome inhibitors may alleviate the clinical manifestations of APS by reducing inflammatory mediators (54, 55). These results indicate that targeting the UPS could represent a novel therapeutic strategy for controlling the inflammatory and thrombotic processes associated with this condition.

Expanding the scope of autoimmune interactions, Duan et al. employed bidirectional Mendelian randomization to elucidate causal relationships between psoriasis, psoriatic arthritis, and multiple autoimmune diseases, including systemic lupus erythematosus (SLE), Crohn’s disease (CD), Hashimoto’s thyroiditis (HT), RA, vitiligo, and AS. Their analysis revealed that CD and vitiligo increase the risk of developing psoriasis (PsO), whereas bullous pemphigoid appears to reduce it. For PsA, risk factors extended to CD, HT, RA, AS, SLE, and vitiligo. These results underscore the interconnectivity of autoimmune disorders and highlight the importance of carefully monitoring for disease progression, particularly in patients presenting with coexisting autoimmune conditions. This study exemplifies how genetic epidemiology can contribute to risk stratification and early intervention strategies, guiding personalized patient management. Proteome-wide analyses complement genetic studies by identifying causal proteins that may serve as biomarkers or therapeutic targets.

Zhao et al. performed a Mendelian randomization study examining 1,837 plasma proteins in relation to PsA risk. They identified seven proteins associated with disease susceptibility, notably interleukin-10 (IL-10), which is inversely linked with PsA, and apolipoprotein F (APOF), which is positively associated with the disease. Colocalization analyses confirmed genetic overlap with disease risk, while phenome-wide assessments suggested broader systemic effects. These findings provide novel insights into PsA etiology and highlight IL-10 and APOF as potential targets for therapeutic intervention, bridging the gap between molecular discovery and clinical translation.

Similarly, in AS, a chronic immune-mediated arthritis with an incompletely understood pathogenesis that primarily affects the axial joints (56–58), Zhao et al. identified eight plasma proteins causally associated with disease risk, including AIF1, TNF, FKBPL, AGER, ALDH5A1, and ACOT13. Colocalization analyses confirmed these as shared causal variants, while phenome-wide assessments highlighted potential adverse effects, offering guidance for drug development. Together, these studies illustrate the power of multi-omics approaches in elucidating the molecular mechanisms underlying autoimmune diseases and supporting the design of targeted therapies.

The role of the microbiome is emerging as a critical modifier of autoimmune pathogenesis (59–66). Lu et al. reviewed the contributions of the gut and oral microbiota to RA, emphasizing that dysbiosis in the gut—including the expansion of *Prevotella* species—and colonization by oral pathogens, such as *Porphyromonas gingivalis* and *Aggregatibacter actinomycetem comitans*, can promote the production of anti-citrullinated protein antibodies (ACPAs), a hallmark of RA.

Bacterial extracellular vesicles were also highlighted as potent mediators of systemic inflammation, suggesting that microbial communities at mucosal sites can modulate systemic autoimmunity.

Complementing this, Yang et al. demonstrated in a collagen-induced arthritis rat model that oral administration of *Bifidobacterium animalis* BD400 alleviates disease progression by modulating gut microbiota composition, enhancing intestinal barrier proteins, and downregulating histidine metabolites implicated in inflammation. This dual approach—mechanistic understanding of microbial contributions and experimental manipulation—provides compelling evidence for microbiota-targeted interventions as potential preventive or adjunctive strategies in autoimmune disorders. Bridging molecular and clinical perspectives, Liu et al. explored the systemic consequences of RA and revealed a causal association with increased epilepsy risk through Mendelian randomization. This finding underscores the fact that autoimmune inflammation extends beyond the affected joints, necessitating comprehensive patient management that considers neurological comorbidities. Similarly, Guo et al. evaluated therapeutic interventions in severe systemic rheumatic diseases by comparing plasma exchange alone with a combination of IVIG and methylprednisolone pulse therapy. Their retrospective analysis demonstrates that adding IVIG/IVMP does not improve survival or ICU stay but increases infection rates, suggesting that simplified monotherapy may suffice in critical care contexts, reducing complications while maintaining efficacy.

Innovative technological platforms further expand the toolkit for understanding autoimmune pathogenesis. Zhang et al. introduced a synovial joint-on-a-chip model that accurately mimics the joint microenvironment by integrating fluid dynamics, mechanical stimulation, and intercellular communication. This platform facilitates preclinical modeling of RA, enabling precise evaluation of inflammation, drug efficacy, and personalized therapeutic strategies. Coupled with mechanistic and molecular insights from the other studies, such platforms can accelerate translational research, bridging the gap between bench and bedside.

Collectively, these contributions highlight a unifying theme: autoimmunity is a multidimensional process shaped by immune dysregulation, genetic predisposition, proteomic signatures, microbial interactions, and systemic consequences. Across RA, PsA, PsO, AS, and APS, these studies underscore the importance of integrating molecular, microbiological, and clinical data to contribute to risk stratification, biomarker discovery, and targeted interventions.

The convergence of genetic epidemiology, proteomics, microbiome research, and advanced modeling technologies emphasizes that autoimmune diseases are not single-organ pathologies but rather a networked, systemic phenomenon. Furthermore, the research presented in this Research Topic emphasizes translational and clinical implications. Targeted immunotherapies, such as low-dose IL-2 in D2T RA, demonstrate the potential to restore immune balance with precision. Proteomic analyses identify actionable biomarkers and druggable targets in PsA and AS, paving the way for personalized therapeutics. Microbiota interventions show promise for disease prevention or modulation of progression, while organ-on-a-chip platforms provide realistic preclinical models to optimize drug development and predict adverse effects. Together, these advances signify a paradigm shift toward integrated, precision medicine approaches in autoimmune disease management.

Lupus is another chronic autoimmune disease discussed in this Research Topic. It is characterized by dysregulated immune responses that lead to inflammation and immune-mediated injury, which may affect various organs (67–69). There are four original papers dedicated to lupus in this Research Topic. The serological profile of SLE was explored by Nicola et al. and anti-dsDNA antibodies were found to be statistically significant for both malar rash and proteinuria; anti-Ro/SSA antibodies were also found to have an association with photosensitivity and pericarditis; additionally, an association was found between anti-Ro antibodies and proteinuria, but only when anti-dsDNA antibodies were present. A similar study focusing on another circulatory marker, the Myc-induced nuclear antigen (Mina) 53 protein, was evaluated in SLE patients by Zamani et al. The study showed that SLE patients have significant increases in Mina53 serum levels along with Mina53 gene expression. Moreover, Mina53 serum levels and gene expression correlated with SLE disease and its severity. Szabó et al. studied circulating immune cell subsets in SLE. Peripheral T-cells, NK-cells, NKT-cells, B-cells, and monocytes were investigated for their glycosylation patterns, and the authors reported that these alterations correlate with disease severity in SLE, which may have implications for the pathogenesis of this condition. Circulatory neutrophils are important players in SLE (70–72) and were investigated by Wang et al. Their report shows that immune complex-driven RNA-sensing by TLR8 in neutrophils is a major mechanism of neutrophil activation in this systemic autoimmune disease. Moreover, the study emphasizes that patients with elevated neutrophil activation and the presence of RNA-containing immune complexes can undergo therapies that rely on TLR8 inhibition and RNA removal. In their complex study, Kramer et al. have evaluated IgE autoantibodies to nuclear antigens in patients with different connective tissue diseases (CTDs), such as SLE, Sjögren’s syndrome (SS), and mixed connective tissue disease (MCTD). Serum analysis of 342 subjects revealed that IgE anti-SSA/Ro-, -SSB/La-, -RNP-, and -dsDNA antibodies exhibit high frequency and specificity for the evaluated CTDs. Moreover, the authors showed that the investigated antibodies may correlate with disease activity and cutaneous or pulmonary involvement. These results demonstrate the potential value of IgE autoantibodies as biomarkers of disease activity and severity, suggesting new directions for the differential diagnosis and therapeutic monitoring of systemic autoimmune diseases.

The review by Xu et al. examined the implications of sterol regulatory element-binding proteins (SREBPs) transcription factors in the pathogenesis of autoimmune rheumatic diseases, such as SLE, RA, and gout. SREBPs regulate lipid metabolism and cholesterol synthesis, thereby influencing cytokine production, inflammation, and the proliferation of germinal center B (GCB) cells. Dysregulation of these pathways contributes to pathological immune activation and the tissue damage characteristic of these diseases (73–77). Identifying the role of SREBPs in the interaction between metabolism and the immune response opens innovative therapeutic perspectives that aim to control inflammation through the regulation of cellular metabolic processes.

Psoriasis, one of the most common inflammatory skin diseases involving both autoimmune and autoinflammatory mechanisms (78–84), was also presented in our Research Topic with two original papers. Raam et al. described the results of the CRYSTAL study (EUPAS36459), a cross-sectional, retrospective study of Pso adult patients from several Central and Eastern European countries. The patients were evaluated while undergoing treatment with either biological or conventional agents. The Psoriasis Area and Severity Index (PASI), Dermatology Life Quality Index (DLQI), and Psoriasis Work Productivity and Activity Impairment (WPAI-PSO) were evaluated upon therapies. The study showed better disease control in the biological treatment group compared to the non-biological treatment group. Another contribution to our Research Topic regarding psoriasis was the original study by Shi et al., who investigated the role of odd-chain fatty acids (OCFAs)in Pso. The authors found that high plasma levels of total OCFAs were positively associated with white blood cell (WBC) and neutrophil counts. This study highlights that plasma OCFAs may have an immunomodulatory role in immune regulation, disease progression, and comorbidity management in psoriasis.

Other original studies cover various topics in autoimmunity. In a two-sample bidirectional Mendelian randomization study by Yuan et al., reciprocal causality was shown between plasma metabolites and autoimmune diseases. For example, four metabolites were associated with inflammatory bowel disease (IBD), and the highest number of associated metabolites was 37 in type 1 diabetes. The study provides data on discovering new therapeutic targets from the metabolite domain in autoimmunity. The study by Chang et al. investigated the genetic link between inflammatory bowel disease with IBD and conjunctivitis—two frequently associated conditions (85, 86) whose connection remains insufficiently understood from a genetic perspective. Genome-wide association studies (GWAS) and Mendelian randomization methods revealed a significant genomic correlation between IBD and conjunctivitis, limited to chromosome 11. These results support the existence of a shared causal mechanism, reinforcing the genetic basis of immunoinflammatory comorbidities. These findings contribute to a broader understanding of the common etiology of autoimmune diseases and may support the development of integrated diagnostic and therapeutic strategies.

Klekotka et al. conducted a systematic literature review to examine clinical evidence on therapies that aim to restore immune homeostasis in autoimmune diseases such as asthma, atopic dermatitis, RA, SLE, and ulcerative colitis. Their analysis of 26 publications revealed a lack of consensus regarding markers and criteria for assessing immune resolution; however, it identified associations between T-cell regulatory biomarkers and clinical remission. The study highlights the potential of the “immune resolution” concept as a marker of durable remissions, along with the urgent need for methodological standardization in clinical studies.

Pemphigus vulgaris, an autoimmune disease affecting the skin and mucous membranes (87–91), was studied by Zakrzewicz et al. Their original study focused on IgG autoantibodies directed against desmosomal adhesion proteins (e.g., desmoglein-3 and -1), which cause loss of keratinocyte adhesion. Their results show that FcRn (neonatal Fc receptor) binding is necessary for the pathogenicity of recombinant anti-desmoglein-3 antibodies in keratinocytes. The data suggest that the role of FcRn in autoimmune diseases is versatile and cell-type dependent. The report of Hou and Chen described a rare case of pemphigus vegetans, a distinct form of pemphigus characterized by vegetative lesions in intertriginous areas. In this condition, the most common autoantibodies target desmoglein 3 (92, 93). This case underscores the importance of prompt diagnosis and appropriate immunosuppressive therapy, demonstrating the effectiveness of modern therapeutic approaches in treating severe forms of pemphigus.

Severe burn injury can generate autoantigens, and Turan et al. focused their study on the liver-derived selenium (Se) transporter selenoprotein P (SELENOP) as a marker of severe inflammation in the acute post-burn phase. The study presented the presence of SELENOP-aAb correlated with severe burn injury, which could be relevant for severely affected patients.

Our Research Topic also hosts several insightful reviews on this topic. Gong et al. reviewed the hypoxic microenvironment and the role of hypoxia-inducible factor-1 (HIF-1)in RA, SLE, multiple sclerosis (MS), and dermatomyositis (DM). Therapeutic strategies that aim at targeting hypoxic pathways may highlight new avenues for intervention. Immune tolerance is a popular topic in autoimmunity (94–98) and Wixler et al. reviewed the role of small spleen polypeptides (SSPs), which regulate peripheral immune tolerance. For example, SSPs reduced the progression of experimental psoriasis or arthritis in animal models (99). Complex mechanisms triggered by SSPs induce a tolerogenic state in dendritic cells, generating Foxp3+ immunosuppressive regulatory Treg cells. T cells are also the subject of the review by Dwyer et al., but in the context of autoimmune diabetes. Key antigenic T lymphocyte epitopes were identified as contributors to this autoimmune pathology, and the role of islet-specific T lymphocyte populations was also discussed. 

An interesting opinion article by Mustelin and Andrade offered a different perspective on the ‘loss of tolerance’ concept in autoimmunity. The authors discussed four dilemmas regarding loss of tolerance, and their neoantigen hypothesis brought a critical rethinking and re-examination of the current loss of tolerance concept.

The involvement of gut microbiota is a recent and important topic of discussion in autoimmunity (61, 100–109) and Wang et al. reviewed its influence in this domain. The authors showed the complex interplay between the gut microbiota, the host, and the immune system, particularly in diseases such as SLE, RA, Sjögren’s syndrome, T1DM, ulcerative colitis, and Pso (110–125).

The topic of the gut microbiota was also discussed in the contribution by Freuchet et al., which addressed the importance of inflammation and biological variability in synucleinopathies, such as Parkinson’s disease, dementia with Lewy bodies, and multiple system atrophy. This review highlights the central role of neuroinflammation, which is mediated by central nervous system-resident cells, peripheral immune cells, and gut dysbiosis, in triggering and progressing neurodegeneration (126–129). Sex-based differences in prevalence and immune response are also emphasized, with major therapeutic implications. The article supports the need for personalized approaches and specific biomarkers for the diagnosis and tailored treatment of synucleinopathies.

Shi et al. reviewed the role of one type of mesenchymal cell, fibroblasts, in autoimmune diseases and their involvement in dermatological autoimmune conditions such as Pso, vitiligo, and atopic dermatitis. Fibroblast heterogeneity was highlighted in each of these autoimmune diseases, implying new future research directions and possibly new therapeutic targets. Also in the dermatological field, Ungureanu et al. reviewed the autoimmune mechanisms of melanoma (130–132), the most severe form of skin cancer (133), with a very complex pathogenesis (134–140). They emphasized that patients with vitiligo are less likely to develop melanoma (141, 142). Moreover, their article highlighted that drug-associated leukoderma (DAL) is a marker of prolonged disease-free survival in melanoma patients treated with immune checkpoint inhibitors (143, 144).

A more exotic form of autoimmunity, acute non-biliary pancreatitis (ANBP), was investigated in an original study by Anılır et al. Toll-Like Receptor 4 (TLR4) and Toll-Like Receptor 2 (TLR2) gene polymorphisms were studied, and their research findings point to TLR-4 Rs4986790 polymorphism groups that can have diagnostic value in ANBP.

The study by Barzilai et al. investigated the role of vasculitis as a potential marker of disease severity in familial Mediterranean fever (FMF), a genetic autoinflammatory condition (145, 146). A comparative analysis of 27 FMF patients with vasculitis and 100 without vasculitis revealed an association with earlier disease onset, increased severity, higher colchicine doses, and a higher frequency of homozygosity for the M694V mutation. Although vasculitis was not identified as an independent factor of severity, its presence may indicate a more aggressive disease course. The results highlight the clinical value of vasculitis as a monitoring and risk-stratification indicator in the management of FMF patients.

The study by Li et al. explored the role of ferroptosis, a form of cell death dependent on oxidative stress (147, 148), in the pathogenesis of thyroid-associated orbitopathy (TAO), a complex autoimmune inflammatory disease (149, 150). Through bioinformatic analysis of gene datasets and experimental validation, the genes ACO1 and HCAR1 were identified as significant molecular markers, showing reduced expression in the orbital adipose tissue of patients. Correlations with immune cell infiltration suggested a pathogenic mechanism in which macrophages play a key role. These findings provide new insights into the pathophysiological processes underlying TAO and propose ACO1 and HCAR1 as optimal feature genes (OFGs) of ferroptosis, suggesting their potential as diagnostic and therapeutic molecular targets in TAO.

Wang et al. proposed a nomogram-based model to estimate the risk of arteriolar lesions in patients with IgA nephropathy, which is a major cause of chronic kidney disease (151–153). Based on a retrospective analysis of 547 cases, predictive factors such as age, mean arterial pressure, eGFR, and serum uric acid were identified. The model demonstrated good performance (C-index 0.72–0.78) and accuracy in predicting arteriolar damage. This tool provides a simple and reliable method for assessing renal prognosis, enabling early intervention in the management of patients with IgA nephropathy. Also in the nephropathy domain, the review by Zhang et al. synthesized evidence regarding the involvement of the complement system in anti-glomerular basement membrane glomerulonephritis (anti-GBM GN), a rare autoimmune disease that often progresses to end-stage renal disease (154). Prior studies have demonstrated the activation of all three complement pathways and a correlation between complement-related proteins and lesion severity. The identification of biomarkers of complement activation enables risk stratification of renal deterioration and paves the way for the use of complement inhibition as a novel therapeutic strategy (155–159). These findings underscore the importance of complement function assessment in the prognosis and management of patients with anti-GBM GN.

In the therapy domain, the study by Zhang and Sun evaluated the potential of genetically engineered T-cell therapies (CAR-T and CAR-Treg) for treating autoimmune kidney diseases that are refractory to conventional therapies. By reprogramming T cells to target autoreactive B cells or antibody-secreting plasma cells, these therapies can modulate inflammation and prevent tissue damage (160, 161). The review summarizes recent fundamental and clinical research, highlighting the efficacy of precise targeting in immune regulation. These advances open revolutionary therapeutic perspectives in immune-mediated kidney diseases, marking a transition toward personalized cellular medicine.

In conclusion, this Research Topic captures the dynamic landscape of autoimmune research, emphasizing mechanistic understanding, biomarker discovery, and innovative therapeutic strategies. By linking immunology, genetics, proteomics, microbiology, and technology, these studies collectively advance our understanding of autoimmune pathogenesis and offer new avenues for personalized interventions. As the field moves forward, these interdisciplinary approaches will be essential for translating mechanistic discoveries into clinical impact, ultimately improving patient outcomes and fostering the development of novel, targeted therapies for autoimmune diseases.
